# Neoadjuvant endocrine therapy in locally advanced estrogen or progesterone receptor-positive breast cancer: determining the optimal endocrine agent and treatment duration in postmenopausal women—a literature review and proposed guidelines

**DOI:** 10.1186/s13058-020-01314-6

**Published:** 2020-07-20

**Authors:** Lauren I. Madigan, Phuong Dinh, J. Dinny Graham

**Affiliations:** 1 Sydney Medical School – Westmead, The University of Sydney, Sydney, Australia; 2 Present Address: South Eastern Sydney Local Health District, and St. George and Sutherland Clinical Schools, UNSW Medicine, Sydney, Australia; 3 Westmead Breast Cancer Institute, Westmead Hospital, Westmead, Australia; 4 The Westmead Institute for Medical Research, The University of Sydney, Westmead, Australia

**Keywords:** Neoadjuvant endocrine therapy, Selective estrogen receptor modulators, Aromatase inhibitors

## Abstract

**Introduction:**

For patients with locally advanced estrogen receptor or progesterone receptor-positive breast cancer, neoadjuvant endocrine therapy (NET) facilitates down-staging of the tumor and increased rates of breast-conserving surgery. However, NET remains under-utilized, and there are very limited clinical guidelines governing which therapeutic agent to use, or the optimal duration of treatment in postmenopausal women. This literature review aims to discuss the evidence surrounding (1) biomarkers for patient selection for NET, (2) the optimal neoadjuvant endocrine agent for postmenopausal women with locally advanced breast cancer, and (3) the optimal duration of NET. In addition, we make initial recommendations towards developing a clinical guideline for the prescribing of NET.

**Method:**

A wide-ranging search of online electronic databases was conducted using a truncated PIC search strategy to identify articles that were relevant to these aims and revealed a number of key findings.

**Results:**

Randomized trials have consistently demonstrated that aromatase inhibitors are more effective than tamoxifen, in terms of objective response rate and rate of BCS, and should be used as first-line NET. The three available aromatase inhibitors have so far been demonstrated to be biologically equivalent, with the choice of aromatase inhibitor not having been shown to affect clinical outcomes. There is increasing evidence for extending the duration of NET beyond 3 to 4 months, to at least 6 months or until maximal clinical response is achieved. While on-treatment levels of the proliferation marker Ki67 are predictive of long-term outcome, the choice of adjuvant therapy in patients who have received NET and then surgery is best guided by the preoperative endocrine prognostic index, or PEPI, which incorporates Ki67 with other clinical parameters.

**Conclusion:**

This study reveals that in appropriately selected patients, NET can provide equivalent clinical benefit to neoadjuvant chemotherapy in the same cohort, if suitable treatments and durations are chosen. Our findings highlight the need for better defined biomarkers both for guiding patient selection and for measuring outcomes. Development of standard guidelines for the prescribing of NET has the potential to improve both clinical outcomes and quality of life in this patient cohort.

## Introduction

Breast cancer is the most common cancer affecting women, with a one in seven lifetime risk [1]. Each year, approximately two million women are diagnosed with breast cancer worldwide [2, 3]. Estrogen receptor (ER)-positive and/or progesterone receptor (PgR)-positive breast cancer accounts for approximately 70% of all breast cancers, and 85% of those in women over 70 years of age [4–6]. At the time of diagnosis, 7 to 20% of women will present with locally advanced breast cancer (LABC) [7–9]. LABC is defined as tumors that are > 50 mm in size or involve the skin of the breast/chest wall, multiple axillary lymph nodes, or the supra/infraclavicular lymph nodes [10]. The percentage of patients diagnosed with LABC in a particular population is largely dependent of the rate and effectiveness of mammography screening [11, 12]. Data from the USA shows that LABC is more likely to affect racial or ethnic minority groups, in particular African Americans, where 12% are diagnosed with LABC compared to 8% in white American populations [11, 13]. This likely results from health inequities such as poverty, rurality, and reduced rates of health insurance which create barriers in accessing health care programs such as preventative screening [11, 14]. Women under the age of 40 may also be more likely to present with LABC as mammography is not routinely recommended in this group [11]. Similarly, on a global scale, LABC is more likely to be diagnosed in under-developed countries, where rates of LABC can reach up to 60% [11, 12, 15].

Breast cancer, for the majority of patients, is treated with upfront surgery followed by other adjuvant (post-operative) modalities including radiotherapy, chemotherapy, or endocrine therapy [16, 17]. However, for women with LABC, breast-conserving surgery (BCS) is not an option at the time of diagnosis, and there is potential benefit in a neoadjuvant, or pre-operative approach, to chemotherapy or endocrine treatment. The goal of neoadjuvant therapy is to reduce the pre-surgical tumor burden and increase the rate of BCS in mastectomy candidates, or allow operability of a previously inoperable tumor [18, 19]. The advantages of BCS include a reduction in surgical morbidity and mortality and improvement in cosmetic outcomes [17]. Neoadjuvant approaches also provide prognostic and predictive information [18, 20, 21].

Despite ER^+^ breast cancer being the most common subtype of breast cancer [4], clinical guidelines for the neoadjuvant treatment of patients with ER^+^ LABC are inconsistent or lacking in most settings. There are data to suggest that neoadjuvant chemotherapy (NCT) produces the same overall and disease-free survival rate as adjuvant chemotherapy for ER^−^ tumors, with increased rates of BCS [22–25]. However, it is also well recognized that ER^+^ tumors often respond poorly to NCT and therefore require robust alternatives [22–25]. Unfortunately, the adoption of neoadjuvant endocrine therapy (NET) for ER^+^ tumors has been much slower. Three categories of NET are available: selective ER modulators (primarily tamoxifen), selective ER degraders (fulvestrant), and aromatase inhibitors (including letrozole, anastrozole, and exemestane) which block estrogen synthesis [26]. Historically, NET was reserved for patients who were considered too frail or unsuitable for surgery or chemotherapy; however, recent evidence is leading to the expansion of this treatment group [27, 28]. Three to 4 months of NET causes tumor shrinkage in two-thirds of patients and can convert up to 50% of mastectomy candidates into BCS candidates [21, 29]. Despite this, NET in breast cancer has been inadequately investigated and utilized compared to NCT, and the optimal duration of treatment remains unknown [30].

This review examines the application of NET in postmenopausal women with locally advanced ER and/or PgR-positive breast cancer. There are currently limited clinical guidelines outlining which neoadjuvant endocrine agent to use, or the optimal duration of use, in postmenopausal women with ER and/or PgR^+^ breast cancer. In this study, we aimed to investigate biomarkers for determining patient selection and monitoring treatment response, compare outcomes between tamoxifen and aromatase inhibitors, determine the optimal duration of NET, and make initial recommendations as a basis for clinical guideline development.

## Methods

### Search strategy

Multiple online electronic databases (PubMed, MEDLINE, and PREMEDLINE) were searched several times during the study period, the most recent being December 2019, using appropriate keywords and MeSH subheadings (Additional file 1, Table S1). The topic areas identified for the search were *breast neoplasms*, *ER*^*+*^*/PgR*^*+*^*status*, *postmenopausal women*, and *neoadjuvant endocrine therapy*. As pathological complete response is rare in ER^+^ breast cancer, there is often inconsistency in the outcome measures used in studies of NET. Consequently, an outcome measure was not included in the PICO search as this would have excluded a large number of relevant studies. This truncated PIC approach has been previously validated as preferable to the PICO model when conducting a systematic review search, to prevent excluding studies which do not use the specified outcome measure and consequently reducing the sensitivity of the search [31, 32]. The initial search produced 170 papers, of which the abstracts were reviewed to ensure the inclusion and exclusion criteria were met. The reference lists of relevant studies and reviews were used to identify further relevant literature.

### Inclusion criteria

Inclusion criteria include postmenopausal, female, and ER^+^ or PgR^+^ breast cancer.

### Exclusion criteria

Exclusion criteria include metastatic, inflammatory, triple negative, HER2^+^ or male breast cancer, prior breast cancer treatment, recent hormonal therapy, and articles published in a language other than English.

### Limits

There was no cut-off date for literature inclusion, and no restriction on the type of study included in the review as this is a relatively small and under-investigated field and we did not want to limit the scope of the review.

A glossary of terms used and their definitions is given in Additional file 1, Table S2.

No conflict of interest was identified.

## Results

### Biomarkers

In order for neoadjuvant trials to be of value, reliable biomarkers for patient selection and treatment response must be available. It is known that NCT is less likely to result in a pathological complete response (pCR) in ER^+^ tumors compared to ER^−^ tumors, making NET a valuable alternative in these patients [33, 34]. Typically, patients with LABC who are treated with NET will receive post-surgical adjuvant treatment with endocrine therapy with or without chemotherapy. The adjuvant treatment recommendation is guided by the response of the tumor to NET and the estimated risk of recurrence based on the evaluation of the surgical specimen. The German Breast Group NCT trial showed a pCR rate of 22.8% in ER^−^ tumors, compared to only 6.2% in ER^+^ tumors [16, 35]. Rates of pCR for ER^+^ tumors treated with NET are typically as low as 3%; however, this figure may be increased with a longer treatment duration [29]. Consequently, pCR is rarely used as an outcome measure in studies of ER^+^ tumors. A number of different biomarkers exist which will be discussed in the following sections. Biomarkers currently in use for patient selection include high ER or PgR expression and gene expression profiling (low pre-treatment recurrence score (RS) or a high ER pathway activity score). Biomarkers with prognostic value include on-treatment and post-treatment Ki67, the preoperative endocrine prognostic index (PEPI) and P-PEPI, and post-treatment RS.

#### ER/PgR

Patients who have ER^+^ and/or PgR^+^ tumors show markedly improved surgical outcomes with NET compared to ER^−^PgR^−^ tumors [36]. ER expression is commonly reported as an Allred Score, which combines a score for the proportion of positive cells, and an intensity score (Additional file 1. Table S3) [37]. Tumors with an Allred Score of 0–2 are ER^−^, and those with a score of 3–8 are ER^+^ [37]. A study investigating 12 months of neoadjuvant letrozole demonstrated that the likelihood of a positive response to NET increased by 7% for each 1% increase in ER expression [29]. However, analysis of the large P024 trial (4 months of neoadjuvant letrozole vs. tamoxifen) showed that patients with less than 10% ER^+^ cells may still derive benefit from neoadjuvant letrozole, but not tamoxifen, and caution should be observed with a 10% cut-off [16, 36]. Currently, many studies use an ER expression threshold of 50% to try to maximize response, which may be excluding a large proportion of eligible patients [38]. The results of the 2019 St. Gallen conference recommend that any tumor with ≥ 1% ER expression be treated with adjuvant endocrine therapy [39]. Since not all ER^+^ tumors respond to aromatase inhibitors, there is a current need for additional biomarkers to predict patient response [40].

#### Ki67

The Ki67 antigen is an accessible and convenient proliferation marker [41, 42]*.* The P024 trial demonstrated that pre-treatment tumor Ki67 levels were not correlated with relapse-free survival (RFS); however, a lower post-treatment Ki67 level was significantly associated with increased RFS [41]. On or post-treatment Ki67 levels predict relapse risk with greater accuracy than baseline Ki67 levels, whether this is measured at several weeks post-NET initiation (IMPACT trial) or after 3 to 4 months [41]. Additionally, a study of 12 months of neoadjuvant letrozole demonstrated that post-treatment Ki67, along with residual tumor burden, were the only predictors of progression-free survival (PFS) [29]. Ideally, a measurement of the absolute Ki67 level on the surgical biopsy sample would guide treatment decisions; however, the statistical significance of this approach is weaker compared to looking at the percentage decrease in Ki67 [20]. In contrast, a 2018 study assessing biomarkers for predicting response to NET found that Ki67 did not predict complete response or pCR, agreeing with the results of the IMPACT trial [43].

#### Gene expression profiling

Gene expression profiling (GEP) uses multi-gene prognostic signatures that measure expression levels of a defined set of mRNA transcripts in a treatment-naïve breast tumor to provide additional information to existing pathological variables, which enable the accurate prediction of probability of disease recurrence and identify clinically different disease subtypes [44]. GEP is currently used to stratify breast cancer into its intrinsic subtypes: HER-2 enriched, luminal A and B, basal-like and normal-like [45, 46]. Luminal A and B tumors correspond to hormone receptor-positive tumors and are distinguished based on their proliferation status [45]. Accordingly, commercial multi-gene assays are now endorsed by the American Society of Clinical Oncology, St. Gallen/European Society for Medical Oncology and National Comprehensive Cancer Network guidelines, for guiding treatment decisions in the adjuvant setting for ER^+^ breast cancers [3, 39]. More recently, their utility has also been demonstrated for clinical management after neoadjuvant treatment [47, 48].

The recurrence score (RS) is an example of GEP developed primarily to predict distance recurrence of breast cancer in the adjuvant setting [49]. The Oncotype DX 21-gene expression profile assay gives rise to the validated RS between 0 and 100 for women with node-negative ER^+^ breast cancer, representing the risk of recurrence at 10 years [49, 50]. The 10-year risk of distant recurrence for a RS < 18 was 6.8% compared to 30.5% for those with a RS ≥ 31 [42, 51]. A high RS usually involves ER and PR negativity, HER2 positivity, and a high nuclear grade and mitotic count [50]. While a high RS is associated with pCR following NCT, the data for NET did not reach statistical significance [50]. However, it was speculated that patients with a low RS will achieve a greater response to NET [50]. A more recent multi-center phase II trial with neoadjuvant exemestane demonstrated that combining the pre-treatment and post-treatment RS had a greater prognostic value in patients receiving NET in terms of disease-free survival (*p* = 0.0096) while the pre-treatment RS alone did not reach significance [48]. Additionally, the TransNEOS study aimed to evaluate whether the RS could act as a biomarker and predict clinical response to neoadjuvant letrozole and concluded that the post-treatment RS was able to predict clinical response (*p* = 0.009) [52]. Among patients with a RS < 18, 54% achieved a complete or partial response and 79% were candidates for BCS, compared to a higher rate of progressive disease in those with a RS ≥ 31 [52]. A study by Bear et al. used the RS to allocate treatment in patients with ER^+^ tumors who were not candidates for BCS at baseline; with patients with a RS < 11 receiving NET, patients with a RS ≥ 26 receiving NCT and those with a RS from 11 to 25 being randomized to NET or NCT [49]. Rates of clinical response (complete and partial responses) were significantly associated with the RS group; however, rates of successful BCS did not vary significantly between the three groups [49].

Given that most existing multi-gene tests primarily predict risk of disease recurrence and benefit from chemotherapy, they do not directly indicate likely endocrine responsiveness and may not be ideal for patient selection. An assay that estimates ER activity could more accurately predict benefit from NET [47]. Inda and colleagues evaluated an ER pathway activity score, or ERPAS, comprising measurement of the expression 27 “high evidence ER target genes” in pre- and post-treatment tumors and reported that around one-third of ER^+^ tumors had an inactive ER pathway activity score at baseline [47]. Tumors that responded to NET had a higher pre-treatment ERPAS and a greater magnitude of decrease after 2 weeks of treatment [47]. This score may be used to identify the group of patients with ER^+^ tumors who will be non-responders to endocrine therapy [47].

#### PEPI

The randomized P024 trial was used to generate a model to predict relapse risk in patients who receive NET, the preoperative endocrine prognostic index (PEPI) [41]. The PEPI analyzes the post-surgical specimen and combines tumor size (T1/2 vs. T3/4), nodal status (positive or negative), Ki67 level, and ER Allred Score (0–2 vs. 3–8), which were all associated with RFS, to give a score of 0, 1–3, or ≥ 4 (Additional file 1. Table S4) [41]. The scores of 0, 1–3, and ≥ 4 give relapse risks of 10, 23, and 48%, respectively [41]. Clinically, the PEPI is able to identify patients who do not require adjuvant chemotherapy due to low relapse risk [41]. In contrast, patients with a high PEPI should receive all appropriate adjuvant therapies [41]. The risk of relapse is extremely low in patients who receive a PEPI score of 0, as validated by both the P024 and IMPACT trials [26, 53]. In the CARMINA 02 trial, there was a significant increase in relapse risk for patients with a PEPI score ≥ 4, the only variable which was predictive of RFS [28]. NET may be especially important for patients with luminal A tumors, as approximately 25% of these patients have a PEPI score of 0 [54, 55]. PgR, which is a known discriminator of luminal A and B tumors, might strengthen prognostic power when combined with the PEPI score [56]. In a study of 107 post-menopausal patients receiving neoadjuvant exemestane for at least 4 months, pre-treatment PgR positivity > 50% was significantly associated with recurrence-free and cancer-specific survival [57]. When PgR was combined as a binary marker with the PEPI score, the PEPI-P was a significantly stronger predictor of outcome than PEPI alone [57]. It will be important to validate the PEPI-P score in a larger cohort, and overall PEPI validation efforts should continue.

#### Emerging biomarkers

The candidate oncogene *ZNF217* has been recently proposed as a biomarker to assess the clinical response to NET in ER^+^ breast cancer, with high levels of Z*NF217* associated with reduced responses to NET in a prospective study [58]. Interestingly, the low and high *ZMF217* groups were similar at baseline in terms of their Ki67 levels so *ZMF217* is unlikely to be simply a marker of cellular proliferation [58]. A recent study found an association between partial response to NET in ER^+^ tumors and the genes KRAS, CUL2, FAM13A, ADCK2, and LILRA2 [43]. Higher levels of KRAS, MMS19, and IVD were correlated with a PEPI score ≤ 3; however, these genes had limited correlation with Ki67 reduction to ≤ 10% [43].

**Table:** 

*Recommendation*: Patients with a PEPI score of 0 at surgery may be spared adjuvant systemic treatment. Patients with PEPI score > 0 should receive adjuvant therapies as per local oncology practice and the patients’ clinical features. The clinical role of Ki67 is currently unresolved.	

### Choice of neoadjuvant endocrine agent

There have been three large randomized controlled trials comparing tamoxifen to aromatase inhibitors; each demonstrating either superiority or non-inferiority of aromatase inhibitors in the neoadjuvant setting [16, 19, 59, 60]. Tamoxifen is a long-standing treatment for breast cancer; however, it may lead to serious adverse effects including thromboembolism and endometrial cancer [59]. Additionally, tamoxifen resistance is not uncommon, yet approximately one-third of patients with resistance to tamoxifen will still benefit from aromatase inhibition [61]. Aromatase inhibitors block the peripheral conversion of androgen to estrogen, reducing circulating estrogen levels by up to 90% [62].

The P024 trial is the largest trial comparing neoadjuvant tamoxifen and letrozole in postmenopausal women with ER^+^ breast cancer who were ineligible for BCS [16, 59]. Letrozole led to a significantly greater objective response rate (ORR) and rate of BCS (Table 1) [59]. Response rates in the letrozole group were higher than for tamoxifen in all Allred Scores from 3 to 8, demonstrating letrozole’s superiority independently of ER expression level [36]. The most common adverse events in this trial were hot flushes and nausea, which occurred equally with tamoxifen and letrozole (57% of patients in each group) [59].

**Table 1:** Tamoxifen vs. aromatase inhibitors in the neoadjuvant setting

Study	Duration (months)	Dose (mg/day)	No. of patients	Study outcomes	Assessment
P024 Trial Eiermann et al. 2001 [59]	4	Letrozole 2.5 or tamoxifen 20	324	1. pCR: 1.3% letrozole; 1.8% tamoxifen 2. ORR (clinical palpation): 55% letrozole; 36% tamoxifen (*p* < 0.0001). *ER*^*+*^*subgroup:* 60% letrozole; 41% tamoxifen. 3. BCS: 45% letrozole; 35% tamoxifen (*p* = 0.022)	Monthly clinical palpation and ultrasound. Mammography at baseline and prior to surgery.
PROACT Cataliotti et al. 2006 [60]	3	Anastrozole 1 or tamoxifen 20 ± NCT	451	1. pCR: NR. 2. ORR (clinical palpation): 49.7% anastrozole; 39.7% tamoxifen (*p* > 0.5, NET only) 3. BCS: 43% anastrozole; 30.8% tamoxifen (*p* = 0.04, NET only)	Ultrasound and caliper measurements at baseline and 3 months.
IMPACT Smith et al. 2005 [19]	3	Anastrozole 1 or tamoxifen 20 or combination	330	1. pCR: NR. 2. ORR (clinical palpation): 37% anastrozole; 36% tamoxifen; 39% combination (*p* = 0.87) 3. BCS: 44% anastrozole; 31% tamoxifen; 24% combination (*p* = 0.23, *mastectomy at baseline*)	Clinical caliper measurements and ultrasound at baseline, 2, 6 and 12 weeks.
Akashi-Tanaka et al. 2007 [63]	5 (Anastrozole) or 4 (Tamoxifen)	Anastrozole 1 or tamoxifen 20	45	1. pCR: not reported. 2. ORR (clinical palpation): 76.5% anastrozole; 46.4% tamoxifen 3. BCS: not reported.	Monthly clinical assessment.
Semiglazov et al. 2005 [64]	3	Exemestane 25 or tamoxifen 20	151	1. pCR: 2.6% exemestane; 2.7% tamoxifen 2. ORR (clinical palpation): 76.3% exemestane; 40% tamoxifen (*p* = 0.05) 3. BCS: 36.8% exemestane; 20% tamoxifen (*p* = 0.05)	Not reported.

*BCS* breast-conserving surgery, *pCR* pathological complete response, *ORR* objective response rate, *CR* complete response, *PR* partial response, *NR* not reported. See Additional file 1, Table S2 for definitions

Two large trials compared neoadjuvant tamoxifen and anastrozole (Table 1). In terms of ORR, both the PROACT and IMPACT trials demonstrated non-inferiority of anastrozole to tamoxifen [19, 60]. In terms of BCS, the PROACT trial showed a significantly higher rate in patients taking anastrozole; however, this rate did not reach significance in the IMPACT trial (Table 1) [19, 60]. Hot flushes were the most common adverse event on both medications; however, anastrozole had a lower rate of vaginal discharge and thromboembolic events in the IMPACT trial [19]. Additional studies which demonstrate the superiority of aromatase inhibitors in comparison to tamoxifen are summarized in Table 1 [63, 64].

There have also been a number of trials comparing different aromatase inhibitors, or aromatase inhibitors with fulvestrant, a selective estrogen receptor degrader (Table 2) [26]. Overall, these studies have not been able to demonstrate superiority of one aromatase inhibitor over another. In terms of ORR, the largest trial (Z1031 trial) did not demonstrate a significant difference between exemestane (62.9%), letrozole (74.8%), and anastrozole (69.1%) (Table 2) [53, 54]. Other studies comparing aromatase inhibitors also did not demonstrate superiority of any one agent (Table 2) [28, 65, 66]. In terms of the BCS rate, there was no significant difference found between the types of aromatase inhibitors or fulvestrant (Table 2) [28, 53, 54, 65, 66].

**Table 2:** Comparison of aromatase inhibitors and fulvestrant

Study	Duration (months)	Dose (mg/day)	No. of patients	Study outcomes	Assessment
Z1031 trial. Ellis et al. 2011 and 2017 [53, 54]	4	Exemestane 25 or letrozole 2.5 or anastrozole 1	377	1. pCR: 1.6% (*AI 16 weeks*); 5.7% (*switched to chemotherapy at 2 weeks*) 2. ORR (clinical palpation): 62.9% exemestane; 74.8% letrozole; 69.1% anastrozole. 3. BCS: 51% (*patients mastectomy at baseline*); 83% (*patients marginal for BCS*)	Monthly physical examination, toxicity assessment, and tumor assessment.
CARMINA 02 Lerebours et al. 2016 [28]	4 or 6	Anastrozole 1 or fulvestrant 500 mg/month	116	1. pCR: NR. 2. ORR (clinical palpation): 52.6% anastrozole; 36.8% fulvestrant. 3. BCS: 57.6% anastrozole; 50% fulvestrant (*p* = 0.5)	Clinical assessment, ultrasound, and MRI at baseline, 1 month, and 4 months.
Grassadonia et al. 2014 [65]	Mean 5.7	Anastrozole 1 or exemestane 25 or letrozole 2.5	144	1. pCR: 1.4% 2. ORR (clinical palpation): 86.6% (9.6% CR and 77% PR) 3. BCS: 84% (*ineligible for BCS at baseline*)	Caliper measurement at baseline, monthly, and before surgery.
FIRST Robertson et al. 2009 [66]	Until progression	Anastrozole 1 or fulvestrant 500 mg/month	205	1. pCR: NR. 2. ORR (clinical palpation): 35.5% anastrozole; 36% fulvestrant (*p* = 0.947) 3. BCS: NR.	Clinical and radiological tumor assessment every 12 ± 2 weeks until progression.

*BCS* breast-conserving surgery, *pCR* pathological complete response, *ORR* objective response rate, *CR* complete response, *PR* partial response, *NR* not reported. See Additional file 1, Table S2 for definitions

Following the determination that all three aromatase inhibitors showed biological equivalence, the Z1031 trial created an extension study known as Z1031B [53, 54]. In the Z1031B trial, patients who had a Ki67 > 10% after 2 to 4 weeks of NET were switched to chemotherapy in an attempt to increase the rate of pCR [53]. Based on the IMPACT trial and Preoperative Letrozole study, patients with a Ki67 > 10% were found to have a < 2% chance of obtaining PEPI = 0 and, hence, avoiding adjuvant chemotherapy [19, 53, 67]. Of the patients who switched to NCT, only 5.7% (2 patients) went on to achieve a pCR [53]. In patients who had a 2-week Ki67 < 10% and received 16 weeks of NET, the pCR rate was 1.6% and the PEPI = 0 rate was 34.4% [53].

The ALTERNATE trial is a phase III trial currently in recruitment which aims to compare anastrozole, fulvestrant, or their combination and identify women at a low risk of recurrence based on the PEPI [26]. After 4 weeks of treatment, women with a Ki67 > 10% will be switched to NCT, and only women with a PEPI = 0 post-surgery will receive adjuvant endocrine therapy [26].

**Table:** 

*Recommendation:* If suitable for NET, post-menopausal patients with ER^+^ and/or PgR^+^ LABC should receive aromatase inhibitors as first-line neoadjuvant therapy. The choice of aromatase inhibitor should be guided by local clinical practice and has not been shown to affect outcomes.	

### Duration of neoadjuvant endocrine therapy

Despite the advantages of aromatase inhibitors being clearly highlighted in the P024 and Z1031A trials, there is continual limited use by physicians [68]. A study from the USA revealed only 3.1% of women with ER and/or PgR^+^ breast cancer received NET compared to 24.7% who received NCT [69]. However, this figure has slowly increased from 2.3% (2004) to 3.5% (2014), with women treated in academic rather than community centers more likely to receive NET [69]. In addition, this study found women were more likely to receive NET if they were > 60 years old and had tumors with a lower grade or stage III disease [69]. Potential barriers to widespread adoption of NET include the heterogenous nature of patient responses, and the long duration required to achieve a clinical response [53]. The reluctance to introduce NET into clinical practice may also represent the poor response rates seen when treatment is carried out for a duration of only 3 to 4 months [30]. In comparison to NCT, NET has more gradual effect on the tumor and an extended treatment period is usually required in order to appreciate the maximum clinical response [70].

The optimal duration of NET that should be administered to postmenopausal patients with ER^+^ breast cancer has not yet been determined or incorporated into clinical guidelines [18, 27, 71–73]. The majority of NET randomized trials use a treatment duration of 3 to 4 months, which is largely arbitrary and related to historical studies of tamoxifen and chemotherapy [30, 73]. At the 2013 St. Gallen breast cancer conference, 62.2% of panelists supported NET being given until maximal response [74, 75]. An additional 26.7% of panelists supported a duration of 4 to 8 months, while only 11.1% supported the current duration of 3 to 4 months [75]. One of the major concerns of extending NET until maximal response is the risk of disease progression. A study by Carpenter et al. aiming to identify the optimal duration of letrozole therapy had a low progression rate of 6.5% [30]. Similarly, a study of neoadjuvant exemestane showed a 7.7% progression rate at 4 months, increasing to only 8% at 6 months of treatment [76]. Despite the evidence, 4 years on at the 2017 St. Gallen conference, uptake of NET remained suboptimal with the panel stating that “Neoadjuvant ET in postmenopausal women with ER^+^ stage II/III tumors is currently underused, although it shows low toxicity” [68]. Six years later at the 2019 St. Gallen International Breast Cancer Conference, there is extremely limited attention given to the neoadjuvant treatment of ER^+^ tumors and no clinical guidelines or recommendations are made [39].

Recently, there have been several small studies aiming to investigate the optimal duration of NET, only two of which were randomized trials. In the four studies where the duration of NET was extended to 12 months, the ORR’s ranged from 76.8 to 95%, and the rate of BCS was 45 to 87.5% (Table 3) [27, 29, 30, 73]. In contrast, the large PROACT and IMPACT trials, discussed previously, used a duration of 3 months and reported an ORR of only 49.7 and 37%, respectively, with BCS rates of 43 and 44% [19, 60]. A single-center study demonstrating the effectiveness of 12 months of letrozole found that in addition to the high ORR of 88%, 13% of patients achieved a pCR and less than 10% of patients progressed [29]. A second single-center study provided further evidence for increased duration of letrozole treatment, with a pCR rate of 2.5% at 4 months, 5% at 8 months, and 17.5% at 12 months [27]. This indicates that the short duration of conventional NET may be the reason behind the low pCR rate frequently observed [27]. A longitudinal phase IV study demonstrated that the median treatment time to allow BCS in previously ineligible patients was 7.5 months, suggesting the conventional duration of 4 months is not optimal [30]. The proportion of patients who became candidates for BCS increased with time, with 66% of patients suitable after 12 months of letrozole treatment [30]. This was an approximately twofold increase in eligibility for BCS when treatment was extended from 4 to 8 months [30]. An earlier phase II trial from 2012 studied letrozole administration over the period of 4 to 12 months and found that maximal response was achieved in a median time of 4.2 months [73]. However, 37% of patients continued to improve beyond 6 months and achieved a maximal response within 6 to 12 months, with the ORR increasing from 55% at 4 months to 76.8% at the conclusion of the study (Table 3) [73]. Other studies which demonstrate the benefit of extending NET are outlined in Table 3 [71, 72]. Interestingly, in the study by Krainick-Strobel et al., of 5 patients who had no change in clinical palpation at 4 months, 1 went on to achieve a complete response and 2 a partial response at 8 months of treatment with letrozole [72]. A retrospective analysis of data in the USA showed that in women who received NET for 1 to 3 months, 20.6% had their tumors down-staged, compared to down-staging in 34.9% of those who received NET for 12 to 24 months [69].

**Table 3:** Duration of neoadjuvant endocrine therapy

Study	Duration (months)	Dose (mg/day)	No. of patients	Study outcomes	Assessment
Rusz et al. 2015 [29]	12	Letrozole 2.5	42	1. pCR: 14.3% operated cases; 13% overall 2. ORR (clinical palpation): 88% 3. BCS: 45%	Clinical palpation every 3 months. Imaging as necessary.
Carpenter et al. 2014 [30]	Up to 12	Letrozole 2.5	139	1. pCR: not reported. 2. ORR (clinical palpation): 85% (3.2% CR and 81.5% PR) 3. BCS: 66% at 12 months (*all ineligible at baseline*)	Clinical examination and bimodal ultrasound every 2 months until BCS.
Allevi et al. 2013 [27]	Randomized to 4, 8, or 12	Letrozole 2.5	120	1. pCR: 2.5% 4 months; 5% 8 months and 17.5% 12 months. 2. ORR (clinical palpation): 45% 4 months; 86.8% 8 months; 95% 12 months. 3. BCS: 80% 4 months; 85% 8 months; 87.5% 12 months.	Monthly clinical palpation (caliper). Mammography and ultrasound at baseline and before surgery.
Llombart-Cussac et al. 2012 [73]	3 to 12	Letrozole 2.5	70	1. pCR: 0% 2. ORR (clinical palpation): 76.8% (25% CR and 51.8% PR) 3. BCS: 43%	Monthly clinical examination. Mammogram and ultrasound every 8 weeks for first 4 months.
Dixon et al. 2009 [71]	3 to > 24 months	Letrozole 2.5	182	1. pCR: NR. 2. ORR (clinical palpation): 69.8% 3 months; 83.5% > 3 months. 3. BCS: 60% 3 months; 72% > 3 months.	Clinical measurement and ultrasound at 0, 2, 6, and 12 weeks. Mammogram at 0 and 12 weeks. 3 monthly review thereafter.
Krainick-Strobel et al. 2008 [72]	4 to 8	Letrozole 2.5	32	1. pCR: NR. 2. ORR (clinical palpation): 55% 4 months; 72.4% 8 months. 3. BCS: 75.9% *(all ineligible at baseline*)	Monthly clinical examination, ultrasound and mammogram.
TEAM IIA Fontein et al. 2014 [70]	3 to 6	Exemestane 25	102	1. pCR: 0.98% 2. ORR (clinical palpation): 58.7% 3 months; 68.3% > 3 months (*p* = 0.031). 3. BCS: feasibility improved 61.8% to 70.6%.	Monthly clinical palpation. 3 monthly MRI, mammogram or ultrasound.
PTEX46 Hojo et al. 2013 [76]	4 or 6	Exemestane 25	52	1. pCR: 0% 4 months; 4% 6 months. 2. ORR (clinical palpation): 42.3% 4 months; 48% 6 months (*p* = 0.89). 3. BCS: 50% 4 months; 48% 6 months.	Monthly caliper measurement and toxicity assessment. Ultrasound and mammogram if progression suspected.

*BCS* breast-conserving surgery, *pCR* pathological complete response, *ORR* objective response rate, *CR* complete response, *PR* partial response, *NR* not reported. See Additional file 1, Table S2 for definitions

Two studies have investigated the optimal duration of NET using exemestane. Exemestane is an irreversible, steroidal aromatase inhibitor which may have fewer adverse effects on the metabolism of lipids and formation of bone when compared to letrozole or anastrozole [77, 78]. The PTEX46 randomized phase II trial found no significant difference in the ORR at 4 or 6 months and suggested the optimal duration of NET is around 4 months (Table 3) [76]. In contrast, the TEAM IIA trial found a significant increase in ORR from 3 to 6 months (Table 3) and concluded that in patients without progressive disease, extending exemestane treatment from 3 to 6 months improves clinical outcomes without increased toxicity [70].

A concern in recommending extended durations of NET is the challenge in evaluating treatment response, coupled with the possibility of disease progression. Although most studies evaluating primary endocrine treatment have generally compared surgery +/− adjuvant endocrine therapy, with endocrine treatment alone, and therefore represent a different paradigm to NET followed by surgery +/− adjuvant endocrine therapy, it is valuable to note that these studies generally support the safety of NET to at least 12 months if only ER^+^ cases are considered. Reviewed by Macaskill et al. [79] and Pepping et al. [80], it is important to note that most trials of primary endocrine treatment without surgery were not focused on LABC, and earlier trials did not select for ER positivity. Trials that selected on ER positivity found no difference in rates of recurrence and disease-specific outcome [79, 80]. The 20-year follow-up report of the Nottingham study of 153 fit elderly patients with ER^+^ breast cancer < 5 cm, randomized to primary tamoxifen or mastectomy with adjuvant tamoxifen [81], revealed no difference in regional recurrence, metastasis, disease-specific, or overall survival between groups. Moreover, while locoregional control was poorer long-term in the no surgery cohort, there was no difference between surgery and no surgery groups until at least 18 to 24 months [81]. This is in agreement with Willsher who reported a progression rate of 3% at 6 months of primary tamoxifen in a cohort selected for high ER, and just 16% requiring surgery after 3 years [82]. Moreover, partial or complete response at 6 months was a strong predictor of excellent control at 3 years. Mustacchi et al. compared outcomes after primary endocrine therapy with tamoxifen, to surgery followed by adjuvant tamoxifen, in 474 women aged 70 and older [83, 84]. No difference in disease-specific survival was observed between groups after 13 years, and although the progression rate was significantly greater in the tamoxifen only group at 80 months, there was no difference between groups at 12 months [83]. These studies suggest that NET for up to 12 months is safe with close monitoring of tumor burden. Treatment beyond 12 months should be considered with caution.

**Table:** 

*Recommendation:* NET should be continued for at least 6 months. In responders at 6 months, NET should be continued until a maximal clinical response is achieved. The best method to determine “maximal response” in clinical practice is complex and not yet standardized.	

### Future directions

Recent studies looking at combining aromatase inhibitors with novel therapies to overcome endocrine resistance have been promising. Phosphatidylinositol 3-kinase (PI3K) inhibitors, including alpelisib and taselisib, have been combined with aromatase inhibitors in a number of recent trials due to the involvement of PI3K-AKT-mTOR pathway upregulation in endocrine resistance [85, 86]. The large randomized phase II LORELEI trial recruited 334 patients with early-stage ER^+^ breast cancer to letrozole plus taselisib or letrozole plus placebo and demonstrated a significant improvement in ORR in the letrozole plus taselisib group [86].

Of recent interest has been the testing of CDK4/6 inhibitors, as these agents have been shown to enhance the activity of aromatase inhibitors and fulvestrant in advanced disease. The randomized phase II PALLET trial investigated the addition of palbociclib, a CDK4/6 inhibitor, to neoadjuvant letrozole in postmenopausal women with ER^+^ breast cancer [87]. This study found that the addition of a CDK4/6 inhibitor led to a significant reduction in proliferation measured by Ki67, compared to letrozole alone, but no significant improvement in clinical response rates over the 14 week study period [87]. The NeoPAL trial demonstrated that this combination of palbociclib and letrozole produced an almost identical rate of clinical response and rate of BCS compared to patients receiving chemotherapy [88]. In the neoMONARCH study which combined abemaciclib with anastrozole, results have shown significantly reduced levels of Ki67 with the CDK4/6 inhibitor with or without anastrozole, compared to anastrozole alone over 14 weeks of therapy [89]. This study also observed an increase in gene expression associated with immune activation at 2 weeks, but only a modest increase in radiological response rates [89]. The CORALLEEN trial was a randomized phase II trial of 106 patients investigating ribociclib plus letrozole vs. chemotherapy in post-menopausal women with hormone receptor-positive breast cancer of the luminal B subtype [90]. At the end of the 6 months of the CORALLEEN trial the rate of patients achieving a PEPI of 0 was 17.3% with chemotherapy and 22.4% with ribociclib and letrozole [90]. Especially when combining with CDK4/6 inhibitors, NET can achieve high rates of clinical response, even in luminal B patients, as compared with chemotherapy [87, 88, 90]. This is in stark contrast to previous thoughts that NET should be for luminal A patients only. Therefore, the benefit of combination therapies is twofold; firstly, they can increase response in luminal A patients, and secondly, they have expanded the patient population to be considered for NET, sparing the toxicities of chemotherapy.

CDK4/6 inhibitors are approved, in Australia, Europe, and the USA, for treatment of ER^+^ metastatic disease in combination with endocrine therapies. While available for LABC, they are not broadly accessible. Currently, ribociclib and palbociclib are available for use in Australia for patients with LABC; however, they are restricted to patients with disease that is deemed inoperable from the outset. These agents are not options for other locally advanced breast cancers where the aim of NET might be to facilitate more restricted breast-conserving surgery or targeted axillary dissection. Investigational treatment strategies combining other targeted agents (including PI3K inhibitors, mTOR inhibitors, and EGFR inhibitors) with endocrine therapies are currently continuing in various clinical trials. In future trial designs, it would be of interest to study not only other novel drug combinations but also to test these combinations over varying treatment durations. Finally, the observation that adding CDK4/6 inhibitors to NET might stimulate anti-tumor immunity [89, 91], as has been observed with some chemotherapy agents [92, 93], suggests that patients with ER^+^ LABC could see improved benefit from second-line immunotherapy approaches, while avoiding cytotoxic chemotherapies. Future clinical trials combining NET and novel targeted agents would benefit from including this outcome measure.

## Conclusion

This review has shown that in well-selected patients with ER^+^ breast cancer, NET can lead to significant clinical benefit, both oncological and cosmetic. Good evidence exists for recommending first-line aromatase inhibitors for at least 6 months and should be developed into a clinical guideline. Despite the significant number of trials investigating NET, there is continued underutilization of this therapy and limited discussion of NET at the yearly St. Gallen conference compared to other treatment modalities [39]. The 2019 ESMO Clinical Practice Guidelines agreed that aromatase inhibitors are preferable to tamoxifen and recommended a treatment duration of 4–8 months in the neoadjuvant setting [3]. However, they did not give clear recommendations for patient selection for NET, highlighting the importance of robust guideline development in this area [3]. This review has also highlighted the lack of biomarkers for NET, thereby limiting the confidence in selecting appropriate patients and in measuring outcomes. Moving forward, it is imperative to keep a prospective registry of all NET patients and incorporate tissue collection for future biomarker studies. Finally, this review has demonstrated that the escalation of endocrine maneuvers to de-escalate chemotherapy achieves similar oncological outcomes with a better quality of life for patients.

## Supplementary information

**Additional file 1: Table S1.** OVID Medline Search Strategy. **Table S2.** Glossary of Terms and Definitions. **Table S3.** Allred Score. **Table S4**: Preoperative Endocrine Prognostic Index (PEPI) Calculation.

## Data Availability

Not applicable

## Abbreviations

ER: Estrogen receptor
PgR: Progesterone receptor
LABC: Locally advanced breast cancer
BCS: Breast-conserving surgery
NET: Neoadjuvant endocrine therapy
NCT: Neoadjuvant chemotherapy
pCR: Pathological complete response
ORR: Objective response rate
RFS: Relapse-free survival
