# Supplementary material for: Neoadjuvant endocrine therapy in locally advanced estrogen or progesterone receptor-positive breast cancer: determining the optimal endocrine agent and treatment duration in postmenopausal women—a literature review and proposed guidelines
Source: Breast Cancer Res. 2020 Jul 20;22:77. doi: 10.1186/s13058-020-01314-6 (PMC7370425; doi:10.1186/s13058-020-01314-6)
Supplement: Supplementary file 1 — Additional file 1: Table S1. OVID Medline Search Strategy. Table S2. Glossary of Terms and Definitions. Table S3. Allred Score. Table S4: Preoperative Endocrine Prognostic Index (PEPI) Calculation. [file 13058_2020_1314_MOESM1_ESM.pdf]

Supplementary Information

Table S1. OVID Medline Search Strategy

# ▲	Searches	Results	Type
1	breast neoplasms/ or unilateral breast neoplasms/	262225	Advanced
2	receptors, estrogen/ or receptors, progesterone/	42029	Advanced
3	Neoplasms, Hormone-Dependent/	5790	Advanced
4	hormone receptor positive breast cancer.tw.	676	Advanced
5	ER+ breast cancer.tw.	773	Advanced
6	ER positive.tw.	4722	Advanced
7	estrogen receptor positive breast cancer.tw.	902	Advanced
8	ER+.tw.	64855	Advanced
9	(ER adj3 breast cancer).tw.	3461	Advanced
10	2 or 3 or 4 or 5 or 6 or 7 or 8 or 9	96542	Advanced
11	postmenopaus*.tw.	46148	Advanced
12	Postmenopause/	22899	Advanced
13	11 or 12	51022	Advanced
14	neoadjuvant endocrine therapy.tw.	155	Advanced
15	neoadjuvant hormonal therapy.tw.	327	Advanced
16	neoadjuvant.tw.	22113	Advanced
17	preoperative endocrine therapy.tw.	27	Advanced
18	preoperative.tw.	194645	Advanced
19	14 or 15 or 16 or 17 or 18	212835	Advanced
20	letrozole.tw.	2098	Advanced
21	anastrozole.tw.	1467	Advanced
22	exemestane.tw.	1001	Advanced
23	fulvestrant.tw.	1003	Advanced
24	tamoxifen.tw.	19680	Advanced
25	Aromatase Inhibitors/	5732	Advanced
26	Antineoplastic Agents, Hormonal/	15363	Advanced
27	20 or 21 or 22 or 23 or 24 or 25 or 26	35159	Advanced
28	1 and 10 and 13 and 19 and 27	170	Advanced

Table S2 Glossary of Terms and Definitions

**Glossary of Terms and Definitions**	
BCS	Breast conserving surgery. Also referred to as lumpectomy or partial mastectomy.
ER	Estrogen receptor.
LABC	Locally advanced breast cancer. Locally advanced breast cancer is defined as tumors that are >50mm in size or involve the skin of the breast/chest wall, multiple axillary lymph nodes or the supra/infraclavicular lymph nodes [1].
NCT	Neoadjuvant chemotherapy.
NET	Neoadjuvant endocrine therapy. This includes tamoxifen, fulvestrant and aromatase inhibitors (letrozole, anastrozole, exemestane).
ORR	Objective response rate. This is usually calculated according to the Response Evaluation Criteria in Solid Tumors (RECIST) [3]. The 4 RECIST categories are complete response (CR), partial response (PR), progressive disease (PD) and stable disease (SD) [4]. CR is defined as complete disappearance of the tumor and reduction in pathological lymph nodes to <10mm [4]. PR is defined as ≥30% reduction in the tumor [4].
pCR	Pathological complete response. This is defined as complete absence of the cancer from the breast and the regional lymph nodes.
PEPI	Preoperative endocrine prognostic index. Calculation and components are discussed in *Appendix 3*.
PgR	Progesterone receptor.
RFS	Relapse free survival. This refers to the length of time following treatment that the patient remains free of signs and symptoms of the disease.

Table S3: Allred Score

The Allred Score is used to determine the ER status of a tumor and has a sensitivity of 99.4% and a specificity of 99.5% [2].

A table showing the calculation of the ER Allred Score is included for your reference:

**Proportion Score (PS)**	**Observation Estimate**	**Intensity Score (IS)**	**Observation Estimate**
0	No staining	0	No staining
1	1%	1	Weak
2	1-10%	2	Intermediate
3	10-33%	3	Strong
4	33-36%	-	-
5	66-100%	-	-

ER Allred Score = Sum of Proportion Score (PS) and Intensity Score (IS)

**Total Score**	**ER Status of the Tumor**
0-2	Negative
3-8	Positive

Table adapted from Qureshi and Pervez, 2010 [2].

Table S4: Preoperative Endocrine Prognostic Index (PEPI) Calculation

The PEPI is calculated using tumor size, nodal status, Ki67 level and ER status (Allred score) of the post-neoadjuvant therapy surgical specimen [4, 5].

The 3 possible PEPI score categories are 0, 1-3 and ≥ 4 [5]. A table summarizing the PEPI calculation is included below for your reference:

**Tumor Characteristics at Surgery**	**RFS HR**	**PEPI Score Allocation**
**Tumor Size**		
**T1/2**	-	0
**T3/4**	2.8	3
**Nodal Status**		
**Negative**	-	0
**Positive**	3.2	3
**Ki67 Level**		
**0 to 2.7%**	-	0
**>2.7% to 7.3%**	1.3	1
**>7.3% to 19.7%**	1.7	1
**>19.7% to 53.1%**	2.2	2
**>53.1%**	2.9	3
**ER Status**		
**Negative**	2.8	3
**Positive**	0	0

RFS = Relapse Free Survival
HR = Hazard Ratio

Table adapted from Ma et. al, 2015 [4].

The scores from the 4 categories are added to give the total PEPI score and hence determine relapse risk.

Note: The ER status (negative or positive) is determined based on the Allred scoring system which is described in Table S3.

References

1. Yalcin B: Overview on locally advanced breast cancer: defining, epidemiology, and overview on neoadjuvant therapy. Exp Oncol 2013, 35(4):250-252.
2. Qureshi A, Pervez S: Allred scoring for ER reporting and it's impact in clearly distinguishing ER negative from ER positive breast cancers. J Pak Med Assoc 2010, 60(5):350-353.
3. Carpenter R, Doughty JC, Cordiner C, Moss N, Gandhi A, Wilson C, Andrews C, Ellis G, Gui G, Skene AI: Optimum duration of neoadjuvant letrozole to permit breast conserving surgery. Breast Cancer Res Treat 2014, 144(3):569-576.
4. Ma CX, Reinert T, Chmielewska I, Ellis MJ: Mechanisms of aromatase inhibitor resistance. Nat Rev Cancer 2015, 15(5):261-275.
5. Ellis MJ, Tao Y, Luo J, A'Hern R, Evans DB, Bhatnagar AS, Chaudri Ross HA, von Kameke A, Miller WR, Smith I, Eiermann W, Dowsett M: Outcome prediction for estrogen receptor-positive breast cancer based on postneoadjuvant endocrine therapy tumor characteristics. J Natl Cancer Inst 2008, 100(19):1380-1388.
